# American Academy of Dental Science

**Published:** 1869-12

**Authors:** Edward N. Harris


### ARTICLE VI.

### *American Academy of Dental Science.*

By EDWARD N. HARRIS, D.D.S.

The second annual meeting of the American Academy of Dental Science, was held in Boston, September 27, 1869, at the Rooms of the Suffolk District Medical Society, No. 36 Temple Place.

The President, Daniel Harwood, M.D., in the chair.

There was a very good attendance of members present, and in addition, a number of invited guests, dentists, physicians, and other scientific men. The Treasurer, Dr. E. G. Tucker, presented his annual statement, which showed the financial affairs of the Academy to be in good condition.

The following officers were elected for the ensuing year:

*President.*—Daniel Harwood, M. D.

*Vice President.*—E. T. Wilson, M.D.

*Rec. & Cor. Secretary.*—E. N. Harris, D.D.S.

*Treasurer.*—E. G. Tucker, M.D.

*Librarian.*—John Clough, M. D.

*Board of Censors.* { E. G. Tucker, M.D., J. L. Williams, M.D., D. M. Parker, M.D.,

Dr. Joseph H. Foster, of New York, was elected to deliver the next Annual Address, and Dr. E. T. Wilson, of Boston, as substitute. The Censors reported the names of several gentlemen for membership in the Academy and they were duly elected.

After some discusion on the different methods of Filling Teeth. Dr. E. T. Wilson read a valuable paper upon "Dental Science" which was well received.

At four o'clock, the President, Dr. Harwood, delivered the Annual Address, his subject: "The Qualification and Education of a Competent Dentist."

His address was a very able and interesting dissertation, listened to with deep interest, and at its close elicited the hearty applause and approval of the assembly.

A vote of thanks was given to Dr. Harwood, and a copy of his address requested for publication.

At 5 o'clock the Academy adjourned to the Parker House to partake of the Anniversary Dinner, which was served in the usual excellent style; Dr. Harwood presided.

Prayer was offered by the Rev. Edward E. Hale. After doing ample justice to the viands, very pleasant after-dinner speeches were made by Dr. Harwood, Rev. E. E. Hale, Rev. Mr. Hinckly, of Dorchester; Dr. J. H. Foster,of New York; Drs. E. T. Wilson; John Clough, of Woburn; E. N. Harris: E. G. Tucker; W. W. Codman; J. L. Williams; A. T. Emery; H. F. Bishop, of Worcester; D. S. Dickerman, of Taunton, and Messrs C. H. Frothingham, M. L. Tucker and C. P. Wilson,

